# Transmission of Seed and Soil Microbiota to Seedling

**DOI:** 10.1128/mSystems.00446-21

**Published:** 2021-06-08

**Authors:** Aude Rochefort, Marie Simonin, Coralie Marais, Anne-Yvonne Guillerm-Erckelboudt, Matthieu Barret, Alain Sarniguet

**Affiliations:** a IGEPP, INRAE, Institut Agro, University of Rennes, Le Rheu, France; b University of Angers, Institut Agro, INRAE, IRHS, SFR QUASAV, Angers, France; University of North Carolina at Charlotte

**Keywords:** microbiota, transmission, seed, root, stem, soil, *Brassica napus*, microbial ecology, plant microbiota, soil microbiology

## Abstract

The seed microbial community constitutes an initial inoculum for plant microbiota assembly. Still, the persistence of seed microbiota when seeds encounter soil during plant emergence and early growth is barely documented. We characterized the encounter event of seed and soil microbiota and how it structured seedling bacterial and fungal communities by using amplicon sequencing. We performed eight contrasting encounter events to identify drivers influencing seedling microbiota assembly. To do so, four contrasting seed lots of two *Brassica napus* genotypes were sown in two soils whose microbial diversity levels were manipulated by serial dilution and recolonization. Seedling root and stem microbiota were influenced by soil but not by initial seed microbiota composition or by plant genotype. A strong selection on the seed and soil communities occurred during microbiota assembly, with only 8% to 32% of soil taxa and 0.8% to 1.4% of seed-borne taxa colonizing seedlings. The recruitment of seedling microbiota came mainly from soil (35% to 72% of diversity) and not from seeds (0.3% to 15%). Soil microbiota transmission success was higher for the bacterial community than for the fungal community. Interestingly, seedling microbiota was primarily composed of initially rare taxa (from seed, soil, or unknown origin) and intermediate-abundance soil taxa.

**IMPORTANCE** Seed microbiota can have a crucial role for crop installation by modulating dormancy, germination, seedling development, and recruitment of plant symbionts. Little knowledge is available on the fraction of the plant microbiota that is acquired through seeds. We characterize the encounter between seed and soil communities and how they colonize the seedling together. Transmission success and seedling community assemblage can be influenced by the variation of initial microbial pools, i.e., plant genotype and cropping year for seeds and diversity level for soils. Despite a supposed resident advantage of the seed microbiota, we show that transmission success is in favor of the soil microbiota. Our results also suggest that successful plant-microbiome engineering based on native seed or soil microbiota must include rare taxa.

## INTRODUCTION

Plants live in complex associations with a wide variety of microorganisms that can modulate their fitness (1–3). Until now, the plant microbiota has been described as acquired horizontally through different environmental sources, including soil (4), air (5), rainfall (6), and insects (7). However, some members of the plant microbiota are also acquired vertically through vegetative propagation (8) or sexual reproduction via seeds (9). Seed microbiota is acquired through different pathways, including horizontal pathways (from the environment) and vertical pathways from the mother plant of the earlier generation. The assembly of seed microbiota is mainly driven from the horizontal pathways (10). So, the initial seed microbiota at sowing is the result of the different processes of acquisition and transmission. The relative contribution of seed microbiota to plant microbiota assemblage has been barely documented because of its recent consideration (9). In spermatophytes, the seed-associated microbial community constitutes the initial inoculum for the next plant generation (11). The seed microbiota can have a crucial role for crop installation by modulating dormancy (12, 13), germination (14, 15), seedling development (16–18), and recruitment of plant symbionts (19). Seed transmission of some specific plant-beneficial or phytopathogenic microbial strains is well documented (20–24). However, little knowledge is available on the fraction of the plant microbiota that is acquired through seeds. Pioneer studies indicate that a fraction of seed microbiota persists during germination and emergence and actively colonizes seedlings (25–27). Still, most of these studies have been carried out under gnotobiotic conditions in the absence of other environmental sources, such as soil.

To date, seed or soil microbiota have been extensively studied in isolation, but their encounters and the assembly mechanisms at play remain broadly unexplored. Seed and soil encounters present a singular situation if the seedling community is considered the outcome community. The initial communities can contribute quite equally to the resultant seedling community or one initial community can become dominant if considering the prevalence of the members. In the latter case, the resident community is generally favored at the expense of the invasive community. This resident advantage can be explained by (i) a higher population size of the resident community (i.e., mass effects) (28), (ii) prior use of resource and space (i.e., priority effects) (29), or (iii) local adaptation of the resident community to the reception niche (i.e., community monopolization effect) (30).

The seed community is extremely reduced in terms of abundance compared to the soil community, but its members have potentially a “resident advantage” (31) as they have been selected by the plant. We therefore hypothesized that despite its lower microbial richness and diversity, seed microbiota being already present and more adapted to the plant environment would benefit from priority effects over soil microbiota for the colonization of plant compartments (19). The plant selection effect can also be reinforced by a plant host genotype contribution. Indeed, the plant genotype together with the cropping environment is a significant driver of seed microbiota composition in Brassica napus (32). Differently, domestication, plant polyploidization, and evolution during cultivation seem not to entail a modification of the overall seed-associated microbial diversity of wheat but to influence diversities and compositions of seed-derived microbial colonizers of axenic wheat seedlings (33).

Nevertheless, adaptation to seeds probably does not recover all features for adaptation to a growing seedling. The initial soil characteristics, including microbial diversity level, are probably other key drivers of seedling microbiota and the persistence of seed-borne microbial taxa. Indeed, we expect that a soil of higher microbial diversity would reduce colonization of the seed microbiota on seedlings due to stronger competition and higher functional redundancy compared to that of a soil of lower diversity.

In the present work, we monitored the outcome of seed and soil communities’ encounter during seedling growth. More specifically, we investigated the following questions: (i) what is the main source of microbial transmission to seedlings, (ii) does seed microbiota composition and soil characteristics, including microbial diversity, impact the assembly of seedling microbiota, and (iii) does the initial taxon abundance in the source influence its transmission success? To answer these questions, we selected seed lots of different genotypes of the winter oilseed rape Brassica napus, which harbored contrasting seed microbiotas (32). We simulated a total of eight different encounter events by sowing the four seed lots in two different soils whose microbial diversity levels were manipulated by serial dilution and recolonization. Bacterial and fungal community structures were characterized by amplicon sequencing of the source seed and soil communities and of the roots and stems sampled at different seedling growing stages. This study gained new insights into the drivers that influence the assembly of the plant microbiota during the early stages of its development.

## RESULTS

### Selected seed lots have distinct microbial communities.

We first characterized the structure and diversity of the source seed communities used in the encounter experiments that originated from two plant genotypes harvested at two different years. Harvesting year and plant genotypes are significant drivers of the diversity structure of the *B. napus* seed microbiota (32). Two *B. napus* genotypes (Boston and Major) collected during two harvest years (Y1 and Y2) were selected for this work. The most prevalent seed-borne bacterial amplicon sequence variants (ASVs) were affiliated with *Sphingomonas* and *Frigoribacterium* genera (see Fig. S1A in the supplemental material). Fungal communities were dominated by ASVs affiliated with *Cladosporium* and *Alternaria* genera (Fig. S1B).

10.1128/mSystems.00446-21.1 FIG S1 Top 5 most prevalent bacterial (A) and fungal (B) ASVs by compartment and presence in the other compartments. Download FIG S1, DOCX file, 0.2 MB. Copyright © 2021 Rochefort et al. 2021 Rochefort et al. https://creativecommons.org/licenses/by/4.0/ This content is distributed under the terms of the Creative Commons Attribution 4.0 International license.

**FIG 1:**
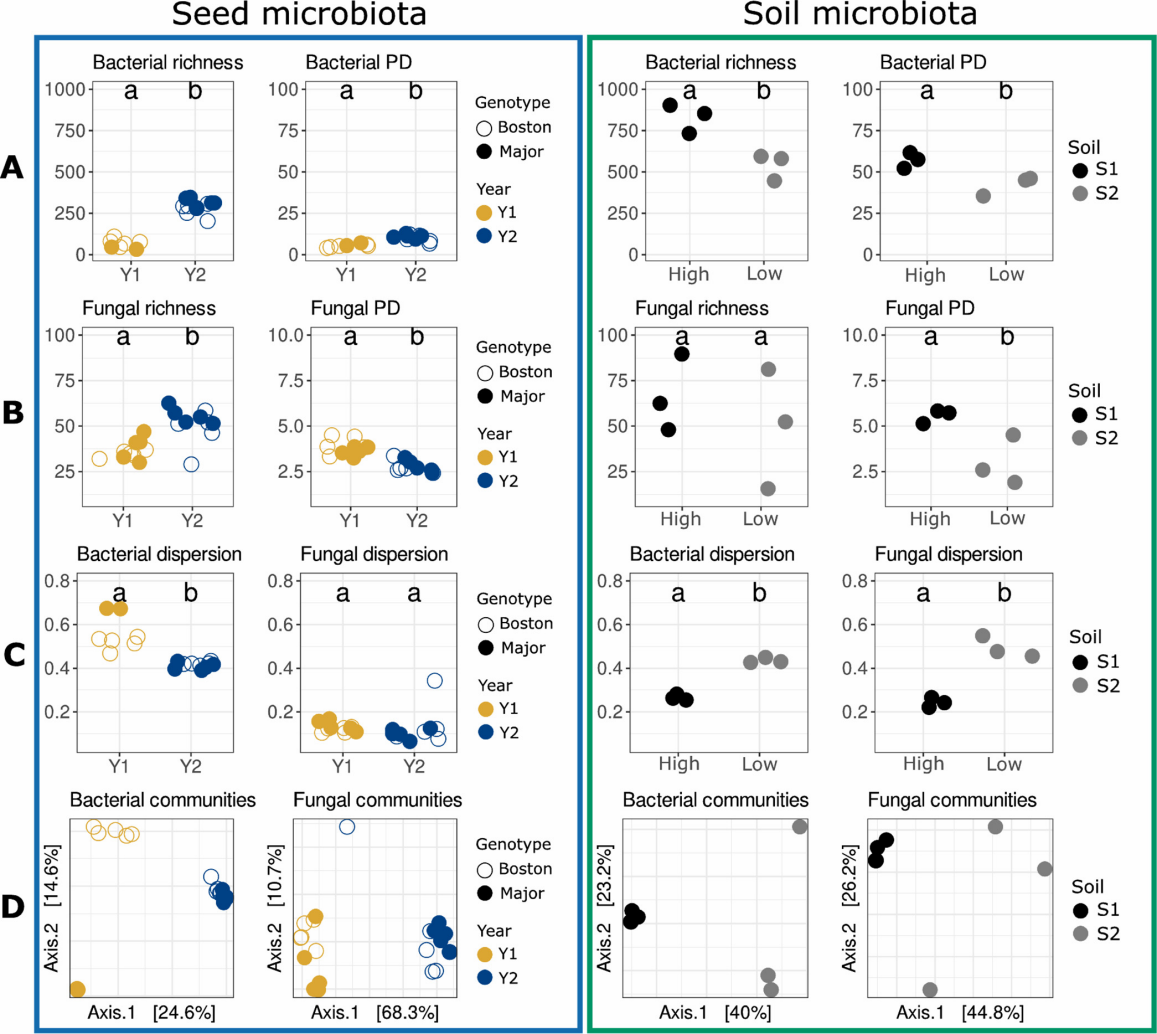
Diversity of initial seed and soil microbial community pools before encounter. (A) Bacterial alpha-diversity. (B) Fungal alpha-diversity. (C) Microbial dispersion to centroid. (D) Microbial beta-diversity. Bacterial and fungal richness were estimated with Chao1. Bacterial and fungal phylogenetic diversities were calculated with Faith’s phylogenetic diversity (PD). Differences in alpha-diversity estimators were assessed with analysis of variance. Differences were considered significant at a *P* value of <0.05. PCoA ordination of the Bray-Curtis index was calculated for bacteria and fungi. Seed microbiota (blue frame, left) was described in two *B. napus* genotypes (Boston and Major) harvested during two consecutive years (Y1 and Y2). Soil microbiota (green frame, right) was described in two soils originating from dilution-extinction serials (S1 and S2).

Estimated bacterial richness (Chao1) was on average at least two times higher in Y2 (250 ASVs) than in Y1 (100 ASVs) for both genotypes, and phylogenetic diversity (Faith’s phylogenetic diversity [PD]) was also significantly (*P* < 0.05) higher in Y2 (Fig. 1A).

While estimated fungal richness was also higher in Y2 (∼50 ASVs) than in Y1 (∼35 ASVs), phylogenetic diversity was higher in Y1 (Fig. 1B). According to distance from the centroid, variation in bacterial and fungal community compositions (Bray-Curtis index) was significantly lower (*P* < 0.05) in Y2 than in Y1 (Fig. 1C). The structure of microbial communities was significantly (*P < *0.001) impacted by harvesting year, with 24% and 62% of the variance explained by this factor for bacteria and fungi, respectively (Fig. 1D). Moreover, seed genotype also significantly (*P* < 0.001, 13% of variance) impacted bacterial community composition but not fungal community composition (Fig. 1D). In brief, the structures of microbial communities were different between the seed lots selected for this study, and harvest year was the most important driver of these changes.

### Manipulation of soil diversity.

To obtain two soils with contrasting levels of microbial diversity, gamma-irradiated soil was inoculated with undiluted and diluted soil suspensions (see Materials and Methods). After 39 days of incubation, a plateau of 10^9^ bacterial CFU and 10^5^ fungal CFU per g of soil was reached for each soil (see Fig. S2). The final bacterial and fungal recolonization levels and the total amounts of extracted DNA were similar in soils S1 and S2 (data not shown). These two endpoints are considered proxies for soil microbial biomass. Therefore, we estimate that the microbial biomasses were similar for both soils. The impact of soil diversity manipulation was assessed on a range of physicochemical parameters. The acidity level of soil S2 significantly (*P* = 0.003) increased by 0.65 pH units compared to that of soil S1. In addition, the concentration of ammoniacal nitrogen was significantly (*P* < 0.001) lower in soil S1, whereas the concentration of nitric nitrogen was twice as large in soil S1 (*P* < 0.001) (Table 1). The relative abundance of ammonia-oxidizing bacteria (AOB) was significantly (*P* < 0.08) higher in soil S1 than in soil S2. Moreover, no nitrite-oxidizing bacteria (NOB) were detected in soil S2, while soil S1 was composed on average of 0.25% NOB.

**TABLE 1:** Soil physicochemical analyses and relative abundance of bacterial nitrifiers^*a*^

Soil	pH or *P* value	Amount (g/kg) or *P* value			C/N ratio or *P* value	Total CaCO_3_ (g/kg) or *P* value	Amount (mg/kg) in one-tenth extract or *P* value for:^*b*^		Percentage^*c*^ or *P* value:	
		Organic C	Total N	Organic material			Nitric nitrogen	Ammoniacal nitrogen	AOB	NOB
S1	6.24 ± 0.03	7.30 ± 0.32	0.84 ± 0.04	12.6 ± 0.56	8.65 ± 0.20	<1	72.2 ± 0.44	1.96 ± 0.42	5.7 ± 2.7	0.25 ± 0.03
S2	6.89 ± 0.09	7.26 ± 0.17	0.84 ± 0.02	12.6 ± 0.31	8.61 ± 0.05	<1	29.03 ± 1.68	32.57 ± 0.93	0.5 ± 0.11	0
										
*P* value^*d*^	0.003*	0.87	1	0.93	0.73	1	0.0002*	2.97.10^−5^*	0.08*	0.005*

a Values for soils S1 and S2 are the averages from 3 replicates ± standard errors.

b Nitric nitrogen, N from NO_3_; ammoniacal nitrogen, N from NH_4_.

c AOB, ammonia-oxidizing bacteria; NOB, nitrite-oxidizing bacteria.

d Data were assessed with *t* tests; significant values are indicated with an asterisk.

10.1128/mSystems.00446-21.2 FIG S2 Temporal soil recolonization by bacteria and fungi. Bacterial and fungal populations are expressed as log10 CFU per gram of soil over time. Download FIG S2, DOCX file, 0.03 MB. Copyright © 2021 Rochefort et al. 2021 Rochefort et al. https://creativecommons.org/licenses/by/4.0/ This content is distributed under the terms of the Creative Commons Attribution 4.0 International license.

The most prevalent soilborne bacterial ASVs were affiliated with *Massilia*, *Nitrosospira*, and *Sphingomonas* (Fig. S1A). The most prevalent soilborne fungal ASVs were affiliated with *Mortierella*, *Trichoderma*, and *Exophiala* (Fig. S1B). The detection of ASVs was based on the nonrarefied data.

According to alpha-diversity indexes (Chao1 and Faith’s index), soil S1 presented a higher (*P* < 0.05) bacterial richness (∼800 ASVs) and phylogenetic diversity than soil S2 (∼500 ASVs) (Fig. 1A). With regard to fungal communities, only phylogenetic diversity was greater in soil S1 (Fig. 1B). Variation in microbial community composition was higher for soil S2 than for soil S1 (Fig. 1C). The initial level of dilution employed explained 40% and 39% of the variance observed in bacterial and fungal communities, respectively (Fig. 1D). Hence, the dilution approach employed in this work resulted in two different soils with contrasting levels of phylogenetic diversity, distinct community structure, pH, and nitric and ammoniacal nitrogen amounts.

### Drivers of seedling microbiota structure.

The outcome of community encounter of seed and soil microbiota was investigated on two plant compartments (stem and root) collected at two distinct plant developmental stages (day 7 [d07] and day 14 [d14]). On the seedling roots and stems, the most prevalent bacterial and fungal ASVs presented abundance patterns clearly influenced by soil origin S1 or S2 (e.g., *Devosia* or Fusicolla aquaeductuum) (Fig. S1). Diverse bacterial (e.g., *Afipia*, *Ensifer*, *Fictibacillus*, and *Nocardioides*) and fungal (e.g., *Peziza*, *Fusicolla*, and Metarhizium) genera were abundant on seedling roots. The stems were dominated by bacterial ASVs affiliated with *Bacillus*, *Nocardioides*, *Devosia*, and *Fictibacillus*, while the most prevalent fungal genera were Fusarium, *Fusicolla*, and *Acremonium* (Fig. S1).

**FIG 2:**
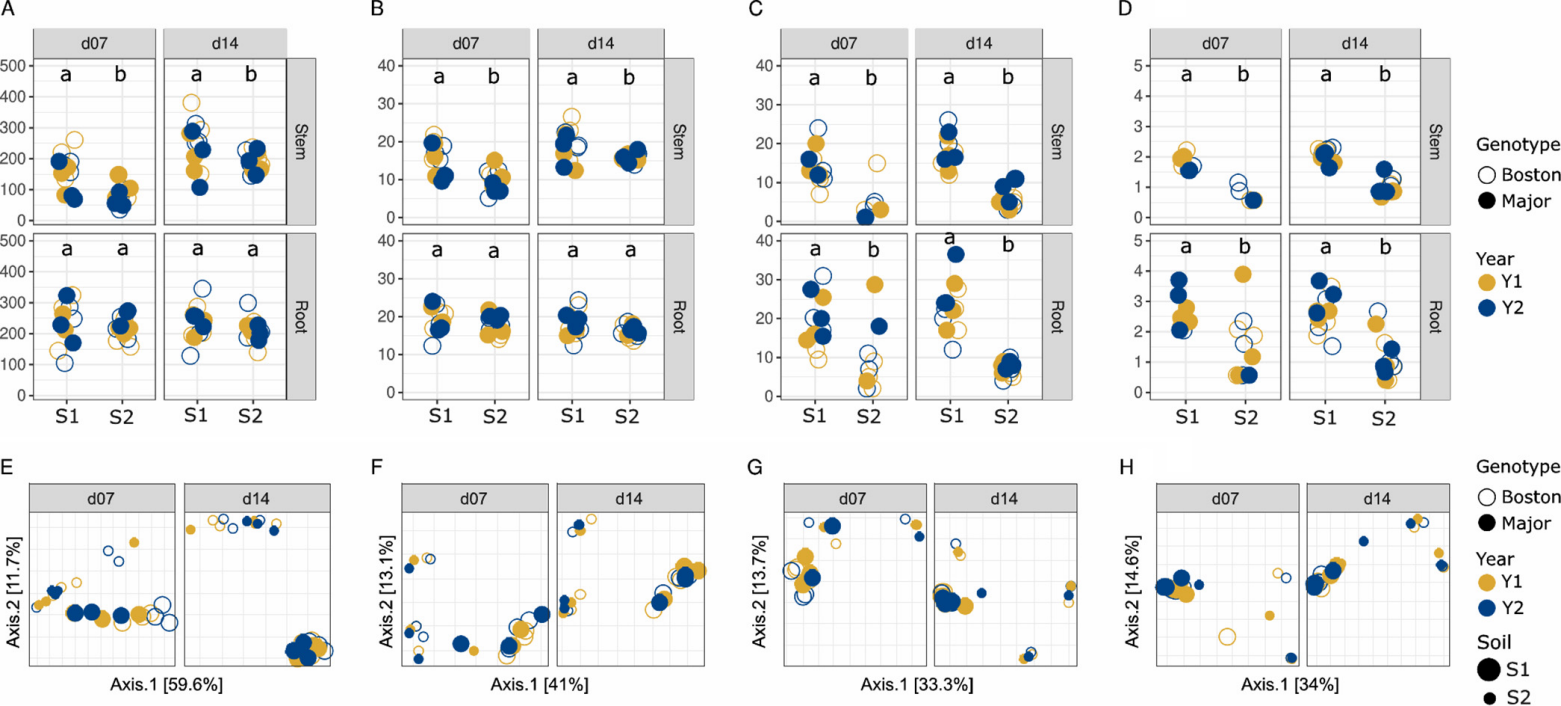
Structures of seedling microbiota after encounter of contrasting seed and soil microbiota. Bacterial estimated richness (A) and phylogenetic diversity (B). Fungal estimated richness (C) and phylogenetic diversity (D). Bacterial beta-diversity in stems and roots (E and F). Fungal beta-diversity in stems and roots (G and H). Open and closed dots are *B. napus* genotypes (A to H). Dots are colored by harvest year (A to H), and dot size represents the soils S1 and S2 (E to H). Bacterial and fungal richness were estimated with Chao1. Bacterial and fungal phylogenetic diversities were calculated with Faith’s phylogenetic diversity. Differences in alpha-diversity estimators were assessed with analysis of variance. Differences were considered significant at a *P* value of <0.05. PCoA ordination of the Bray-Curtis index was calculated for bacteria and fungi.

Seed genotypes and harvest years impacted neither estimated richness nor phylogenetic diversity in stem and root (Fig. 2).

In contrast, soil origin (S1 or S2) significantly (*P* < 0.05) influenced microbial richness (Fig. 2A and C) and phylogenetic diversity (Fig. 2B and D) in stems at d07 and d14, with a higher richness and diversity in stems collected from soil S1 that had a higher initial microbial diversity. No significant change in bacterial richness and phylogenetic diversity was detected in roots between the two soils (Fig. 2A and B), while fungal richness and phylogenetic diversity were almost two times higher in roots from soil S1 at each development stage (Fig. 2C and D).

Moreover, there was a small but significant increase in bacterial richness and phylogenetic diversity in stems at stage d14 compared to that at stage d07. No differences were observed in fungal diversity across stages (Fig. 2).

The composition of the plant microbiota was not influenced by the seed genotype or the harvesting year (Fig. 2E to H and Table 2). In contrast, soil origin S1 or S2 significantly (*P* < 0.001) contributed to the variance observed in stem (14.6%) and root (36.8%) bacterial communities (Fig. 2E and F) as well as the variance in stem (24.1%) and root (22.1%) fungal communities (Fig. 2G and H and Table 2).

**TABLE 2:** Relative influence of soil, genotype by year, and stage factors on seedling root and stem microbial beta-diversity^*a*^

Variable	Bacteria		Fungi	
	*P* value	% of explained variance	*P* value	% of explained variance
Root				
Soil	0.001*	**36.8**	0.001*	**22.1**
Stage	0.001*	**10.2**	0.003*	**8.0**
G×Y	0.955		0.999	
Soil-G×Y	0.990		0.996	
Stem				
Soil	0.001*	**14.6**	0.001*	**24.1**
Stage	0.001*	**46.5**	0.002*	**8.6**
G×Y	0.394		0.986	
Soil-G×Y	0.695		0.878	

a Permutational multivariate analysis of variance of beta-diversity was conducted with a linear model built with the adonis2 function on data separated by root or stem compartment, integrating soils (S1 and S2), sampling stages (d07 and d14), G×Y (interaction between plant genotype and year), and interaction between soil and G×Y. Significant values and their associated percentage of variance (*R*^2^) are in bold and followed by an asterisk (*P* < 0.05).

Since soils S1 and S2 differed in microbial diversity but also in physicochemical properties, we assessed the relative influence of these factors on seedling microbiota structure (Table 3). Soil bacterial richness was the most important driver of microbial community composition associated with roots, with 34.3% and 22.3% of variance explained by this variable for the bacterial and fungal fractions, respectively. The other soil parameters (pH and NH_4_^+^ and NO_3_^−^ contents) also significantly influenced root-community composition with the exception of NO_3_^−^ concentration for the fungal community. The percentage of explained variance was, however, less important for these soil parameters (from 3.4% to 16.6%) than for bacterial richness (Table 3). With respect to stem microbial communities, bacterial richness was also an important driver of bacterial (12.2%) and fungal (20.3%) community composition together with pH (17.3%) for the fungal fraction (data not shown). Of note, the sampling stage was a significant driver for root and stem microbial communities and was especially important for driving bacterial communities associated with the stem. The main difference observed with microbial composition (Faith’s PD) was the relative importance of soil fungal community composition (14%) in bacterial root-community composition (Table 3).

**TABLE 3:** Relative influence of initial soil microbial and physicochemical characteristics, genotype by year, and stage factors on seedling root and stem microbial beta-diversity^*a*^

Group	Parameter	Result for bacteria		Result for fungi	
		*P* value	% explained variance	*P* value	% explained variance
Microbial richness					
Root	Bacterial richness	0.001*	**34.3**	0.001*	**22.3**
	Fungal richness	0.001*	**8.7**	0.001*	**7.1**
	pH	0.001*	**8.1**	0.001*	**16.6**
	NO_3_	0.002*	**3.5**	0.102	1.9
	NH_4_	0.001*	**6.8**	0.006*	**3.4**
	Stage	0.001*	**10.3**	0.001*	**7.6**
	G×Y	0.412	2.0	0.770	2.2
Stem	Bacterial richness	0.001*	**12.2**	0.001*	**20.3**
	Fungal richness	0.006*	**3.8**	0.001*	**8.5**
	pH	0.026*	**3.0**	0.001*	**17.5**
	NO_3_	0.165	1.2	0.004*	**3.8**
	NH_4_	0.062	2.7	0.001*	**5.8**
	Stage	0.001*	**46.5**	0.001*	**6.8**
	G×Y	0.990	1.2	0.770	2.5
Diversity					
Root	Bacterial Faith’s PD	0.001*	**31.9**	0.001*	**19.6**
	Fungal Faith’s PD	0.001*	**14.3**	0.001*	**8.2**
	pH	0.001*	**7.2**	0.001*	**10.1**
	NO_3_	0.001*	**5.7**	0.001*	**11.6**
	NH_4_	0.008*	**2.4**	0.131	1.8
	Stage	0.001*	**10.3**	0.001*	**7.6**
	G×Y	0.437	2.1	0.884	2.2
Stem	Bacterial Faith’s PD	0.001*	**11.2**	0.001*	**17.0**
	Fungal Faith’s PD	0.005*	**4.5**	0.005*	**14.0**
	pH	0.001*	**3.2**	0.001*	**8.9**
	NO_3_	0.042*	**2.2**	0.042*	**12.7**
	NH_4_	0.214	2.7	0.008*	**3.3**
	Stage	0.001*	**46.5**	0.001*	**6.8**
	G×Y	0.280	2.1	0.783	2.5

a Permutational multivariate analysis of variance of beta-diversity was conducted with a linear model built with the adonis2 function on data separated by root or stem compartment, integrating soil (S1 and S2) microbial richness and diversity (Faith’s PD), soil pH, NO_3_ and NH_4_ content, sampling stages (d07 and d14), and G×Y (interaction between plant genotype and year). Significant values and their associated percentage of variance (*R*^2^) are in bold and followed by an asterisk (*P* < 0.05).

### Relative transmission of seed-borne and soil taxa to seedling microbiota.

**FIG 3:**
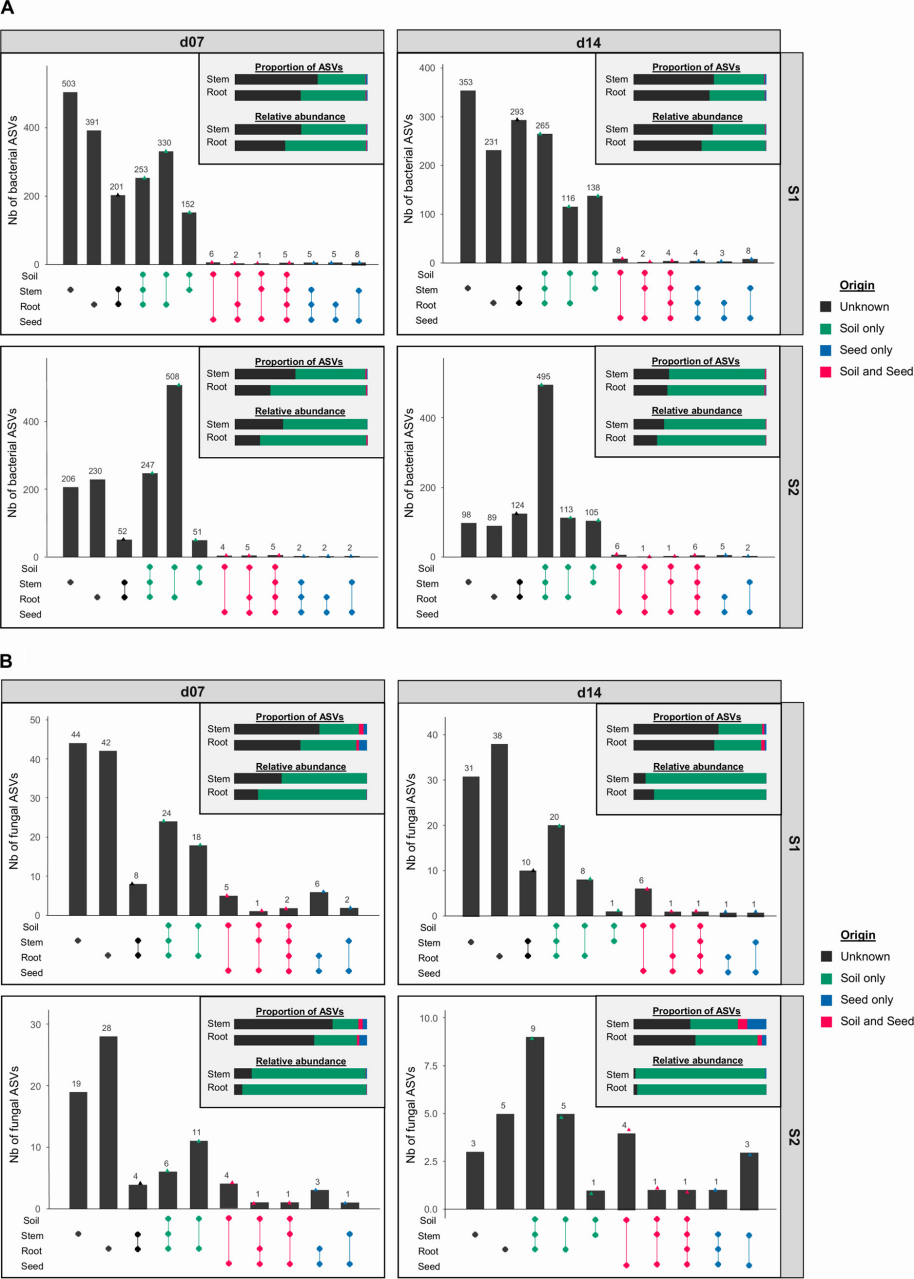
Origin of microbial taxa within stem and root assemblages. Bacterial (A) and fungal (B) ASVs shared between seeds, soil, roots, and stems at each harvesting stage (d07 or d14) and soil (S1 or S2). For each plot, top right bar chart summarizes the proportion and relative abundance of plant-associated ASVs that were detected in soil and/or seeds or not detected (unknown) within these habitats. Compartments (soil, seed, root, and stem) associated with each particular intersection are highlighted with connected dots: in soil roots and/or stems (green), in seeds, roots, and/or stems (blue), in soil, seeds, roots, and/or stems (red), and only in roots and/or stems (unknown origin, black).

To describe the outcome of the community encounter of seed and soil microbiota during seedling growth, we characterized the proportion of bacterial and fungal ASVs from seed, soil, or unknown origin that composed the stem and root seedling microbiota. A total of 39 bacterial ASVs and 16 fungal ASVs were shared between seeds, roots, and stems (Fig. 3). Among these ASVs, 16 bacterial and four fungal ASVs were also found in soil samples.

Between 35% and 72% of seedling-associated bacterial ASVs were also detected in soil (S1 and S2), while these numbers were lower for fungal ASVs (20% to 45%) (Fig. 3). These soil-derived ASVs represented between 40% and 98% of the total seedling microbiota. On the contrary, few seedling-associated ASVs were detected in seeds (0.3% to 15%), representing less than 1% of the microbial relative abundance in roots and stems (Fig. 3). Lastly, a fraction of the seedling microbiota was of undetermined origin as undetected in the initial seed and soil pools. ASVs of undetermined origin either may correspond to unsampled taxa in soil or seed compartments (i.e., rare taxa) or may derive from other environmental sources. The proportion of bacterial ASVs of undetermined origin was higher for plants grown in soil S1 (approximately 45% to 65% of community membership and composition) than those in soil S2 (20% to 30%) (Fig. 3 and Fig. S3A). This result suggests that seedlings are primarily colonized by rare soil taxa that were initially undetected. In contrast, fungal ASVs not detected in soil and seeds represented approximately 40% to 60% of community membership in plants, but their impact on community (i.e., their relative abundance) was lower, with less than 20% regardless of the soil diversity (Fig. 3 and Fig. S3B).

10.1128/mSystems.00446-21.3 FIG S3 Proportion of bacterial (A) and fungal (B) ASVs of unknown origin in seedlings according to diversity of the initial pool (seed or soil). Nature of the initial pool (seed genotype/year or soil) is mentioned and separated with dashed lines. Colors represent the receiving seedling compartment. Download FIG S3, DOCX file, 0.1 MB. Copyright © 2021 Rochefort et al. 2021 Rochefort et al. https://creativecommons.org/licenses/by/4.0/ This content is distributed under the terms of the Creative Commons Attribution 4.0 International license.

**FIG 4:**
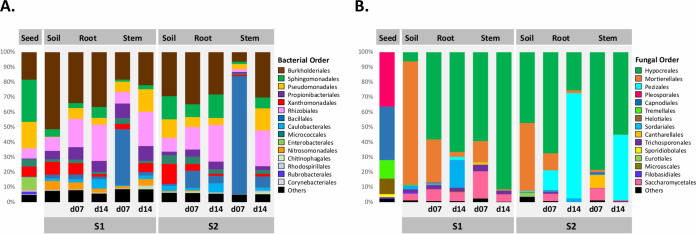
Taxonomic profiles of microbial communities associated with initial *B. napus* seeds and soil and with seedlings at 7 or 14 days after sowing. The 15 most abundant bacterial (A) and fungal (B) orders detected in seeds, soils, roots, and stems are displayed.

Contrasting microbial taxonomic compositions were observed between the initial soil and seed pools. Concerning bacteria, *Enterobacterales* and *Sphingomonadales* were more abundant in seeds, while *Burkholderiales* were more abundant in soils (Fig. 4A). Changes in bacterial order relative abundance were also detected between stem and root, especially at d07, when *Bacillales* dominated the bacterial fraction of the stem microbiota (Fig. 4A). The taxonomic compositions of the fungal fractions of the seed and soil microbiota were highly contrasted at the order level. Seeds were mainly inhabited by Pleosporales, Capnodiales, and Tremellales, when soils principally constituted Mortierellales and Hypocreales (Fig. 4B). The latter order was, however, more prevalent in soil S2 in than in soil S1 (Fig. 4B). As in soil, *Burkholderiales* remained the dominant order in roots but were in part outcompeted by an increasing proportion of *Rhizobiales*. *Xanthomonadales* were reduced in roots, especially in soil S2 and more drastically after 14 days. *Bacillales* were the most dominant order at 7 days in stems before regressing at 14 days. In contrast, *Pseudomonadales* and *Rhizobiales* had a low abundance in stems, especially in soil S2 before increasing at 14 days (Fig. 4A). For fungi, Hypocreales were highly prevalent in seedlings, when Mortierellales were more or less prevalent, at 7 days in roots and stems and then disappeared in roots and were not represented in stems at 14 days (Fig. 4B). These pools represented only a small proportion of transmitted ASVs from seed to seedling compared to that for the pools originating from soil (Fig. 3). Mainly bacterial ASVs belonging to *Bacillales*, *Burkholderiales*, and *Pseudomonadales* and many fungal ASVs belonging to Hypocreales were transmitted from soil to seedlings.

### Seedling transmission success of seed-borne and soil microbiota.

**FIG 5:**
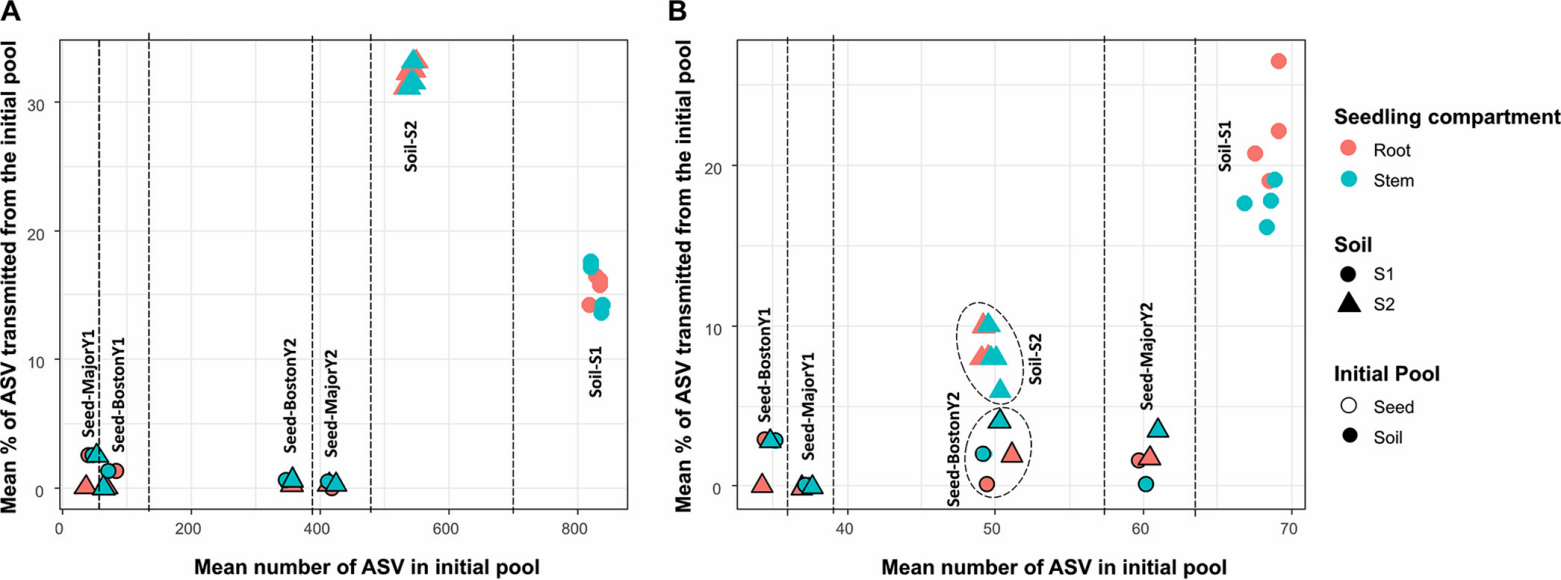
Influence of the initial bacterial (A) and fungal (B) diversity of the colonizing pools on the transmission success to the seedling at d14. The transmission percentage is based on the number of ASVs present in the initial community (seed or soil). Nature of the initial seed pool (genotype and year) or soil type (S1 and S2) is indicated and separated with dashed lines. Colors represented the receiving seedling compartment (root or stem).

The contrasting seed lots and soils employed in the experimental design enabled investigation of the influence of the initial diversity of the colonizing pools on the success of transmission to the seedling (Fig. 5).

For bacteria and fungi, we found no clear relationships between the level of diversity of the initial pool (seed or soil), soil physicochemical characteristics, and the percentage of ASV transmission in seedlings (Fig. 5). The transmission percentage is based on the number of ASVs present in the initial community (seed or soil). Soil microbiota always presented the highest success of ASV transmission to seedlings, with 10% to 30% of the initial ASV pool in soil. Overall, the transmission of soilborne microbiota to seedlings was higher for the bacterial community (113 to 180 ASVs) than for the fungal community (3 to 18 ASVs) (Fig. 5A and B). For bacteria, transmission to seedlings was higher in soil S2 (32% ± 0.84%) than in soil S1 (15.6% ± 1.49%) (Fig. 5A). For fungi, the opposite pattern was observed, with a higher percentage of ASVs transmitted to seedlings from soil S1 (19.8% ± 3.24%) than from soil S2 (8.5% ± 1.41%) (Fig. 5B). A low percentage of ASV transmission from seeds to seedlings was observed for bacteria (0.8% ± 0.93%) and fungi (1.45% ± 1.44%), with no clear influence of the genotype and harvest year (Fig. 5). Finally, overall ASV transmission to seedlings was similar on roots and stems, except for the fungal pool of soil S1, where transmission success in roots was higher (22.1% ± 3.18%) than in stems (17.7% ± 1.2%) (Fig. 5).

### Emergence of rare and intermediate-abundance taxa in seedlings.

**FIG 6:**
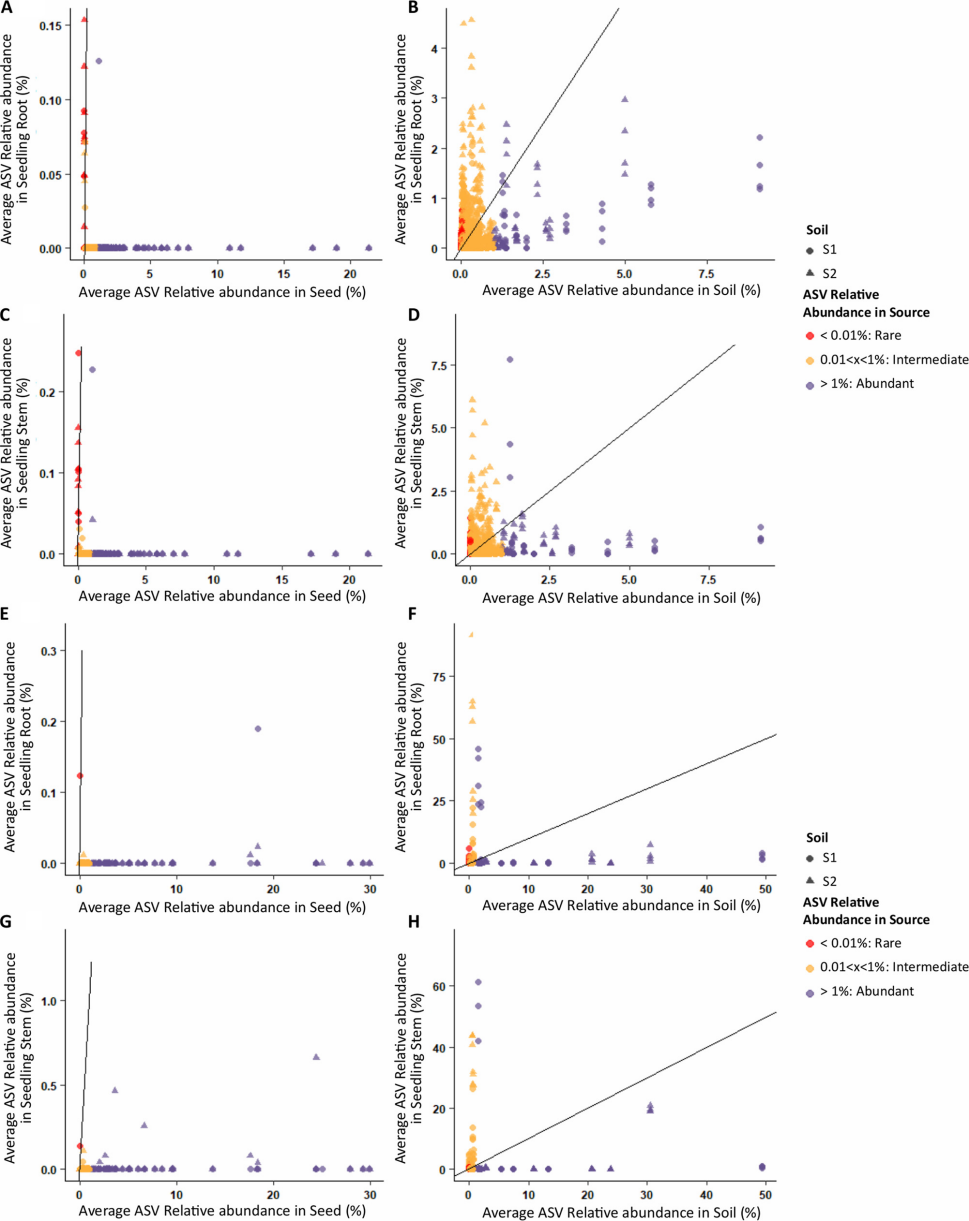
Relationship between relative abundance of transmitted bacterial (A to D) and fungal (E to H) ASVs in the source (seed or soil) and in seedling roots and stems. Each dot represents one ASV and is colored according to its relative abundance in the source (seed or soil). In the graphs on the right (soil), a 1-to-1 line is represented to facilitate the visualization of the ASVs that present a higher abundance in seedlings than soils (above the line) or the opposite pattern.

We next examined the influence of the initial ASV relative abundance on its seedling transmission success and the resulting abundance in roots and stems at d14. ASVs were grouped into three arbitrary abundance classes: rare (average relative abundance of <0.01%), intermediate (between 0.01 and 1%), and abundant (>1%) (34, 35). An interesting finding was that abundant ASVs in the source pool (seed or soil) almost never became abundant in roots and stems (below the 1:1 line) or were not even transmitted to seedlings (Fig. 6). The majority of transmitted seed-borne bacterial ASVs were initially rare taxa, with nine rare ASVs of 11 ASVs transmitted to roots and 11 rare ASVs of 16 ASVs in stems (Fig. 6A and C). Only one abundant seed ASV (of 65 in the initial pool) was found to be transmitted to roots and stems (*Afipia*). One intermediate ASV was successfully transmitted to both roots and stems (*Blastomonas*) and three intermediate ASVs to stems solely (Pseudomonas lurida, Cutibacterium acnes, and *Hyphomicrobium*) (see Table S1). It should be noted that none of these abundant and intermediate transmitted seed-borne bacterial ASVs became abundant in seedlings (relative abundance of <0.3%).

10.1128/mSystems.00446-21.6 TABLE S1 Bacterial and fungal ASVs transmitted from seed to seedling (root and/or stem). Relative abundances in seed are as follows: rare, <0.01%; intermediate, 0.01% to <1%; abundant, >1%. Download Table S1, DOCX file, 0.02 MB. Copyright © 2021 Rochefort et al. 2021 Rochefort et al. https://creativecommons.org/licenses/by/4.0/ This content is distributed under the terms of the Creative Commons Attribution 4.0 International license.

The most abundant root bacterial ASVs (>1%) originated from soilborne ASVs classified as intermediate ASVs (*n* = 26 ASVs) and abundant ASVs (5 ASVs) (Fig. 6B and D). For the stems, most of the abundant ASVs originated from intermediate soil ASVs (*n* = 24) along with a few abundant ASVs (*n* = 6) and one rare ASV. When considering the entire seedling bacterial microbiota and not only the most abundant taxa, we found that 95% of the abundant soil ASVs, 40% of the intermediate ASVs, and 28% of the rare ASVs were transmitted to roots. For the stems, 86% of abundant ASVs, 40% of intermediate ASVs, and 29% of rare ASVs were transmitted.

For the fungal community, four seed-borne ASVs were transmitted to both roots and stems, composed of two abundant ASVs (Alternaria infectoria and Cladosporium delicatulum), one intermediate (*Alternaria* sp.), and one rare (Gibberella avenacea). In addition to these four ASVs, three additional abundant ASVs, all affiliated with *A. infectoria*, were transmitted to the stems (Table S2). For soilborne fungal ASVs, the most transmitted taxa to roots were intermediate ASVs (19 ASVs, 12% of initial pool), with a few abundant taxa (nine ASVs, 53% of initial pool) and seven rare ASVs (12% of initial pool) (Fig. 6E and F). Similarly, soilborne ASVs transmitted to stems were predominantly intermediate ASVs (15 ASVs, 10% of initial pool) along with seven abundant ASVs (41% of initial pool) and six rare ASVs (11% of initial pool) (Fig. 6G and H). It should be noted that some of these soilborne fungal ASVs present extremely high relative abundance in seedlings (>20% relative abundance) compared to that of seed-borne ASVs (<2% relative abundance).

10.1128/mSystems.00446-21.7 TABLE S2 Bacterial and fungal ASVs transmitted by the soil and becoming abundant (>1%) in seedling. Relative abundances in soil are as follows: rare, <0.01%; intermediate, 0.01% to <1%; abundant, >1%. Download Table S2, DOCX file, 0.02 MB. Copyright © 2021 Rochefort et al. 2021 Rochefort et al. https://creativecommons.org/licenses/by/4.0/ This content is distributed under the terms of the Creative Commons Attribution 4.0 International license.

The taxonomic information regarding microbial ASVs transmitted to seedlings is reported in Table S1 (seed-borne) and Table S2 (soilborne). Taxa were distributed across the entire phylogeny. For example, *Pseudomonadales* and *Burkholderiales* were distributed in intermediate and dominant taxa. The absence of phylogenetic signal was also observed for fungal ASVs. For instance, Hypocreales were found in rare, intermediate, and abundant taxa. Altogether, these results indicate that seed-borne taxa transmitted to seedlings are predominantly rare taxa, while transmitted soilborne taxa are primarily intermediate and abundant taxa. Moreover, this analysis shows that the transmission rates of fungi are two to four times lower than those for bacteria for all ASV abundance classes (dominant, 41% to 53% versus 86% to 95%; intermediate, 10% to 12% versus 40%; rare, 11% to 12% versus 28% to 29%).

## DISCUSSION

### Outcome of seed and soil community encounter in favor of soil microbiota.

Soil had a great influence on the plant microbiota composition, since 70% and 30% of soilborne bacterial and fungal taxa, respectively, were detected in seedlings. In contrast, transmission of seed-borne microorganisms to roots and stems of *B. napus* was lower than that to soil, with on average 2% of bacterial ASVs and 12% of fungal ASVs detected in seedlings. The remaining seedling-associated ASVs (present in root and/or shoot) were not identified in either seed or soil habitats.

It is highly plausible that most of these taxa were soilborne and were initially not sampled due to the high heterogeneity of soil spatial structure (36). Indeed, there were on average many more bacterial ASVs of undetermined origin in the seedlings growing in the soil of high diversity, lower pH, and higher nitric content (>50%) than in those from a soil of low diversity, higher pH, and higher ammoniacal content (<30%).

### The impact of soil diversity on plant microbiota composition increased over time in seedlings.

The impact of initial soil diversity on plant microbiota composition increased over time in roots and stems, therefore confirming the influence of soil resident time and plant age on root microbiota dynamics (37). The rhizosphere effect on microbiota composition is usually stronger on older plants than observed here on seedling. The relative soil microbiota selection is due to root exudates, for which amounts increase and the composition changes during root growth (38, 39). Our observations were recorded on early seedlings (07 and 10 days) when the plant effect is weak. For instance, the rice root-associated microbiota only stabilizes after 8 to 9 weeks after germination. Major shifts in the microbiome (i.e., on rice roots) are registered later in correlation with rates of developmental transitions such as juvenile and adult life stages (40).

Weak transmission of seed-borne microorganisms to the rhizosphere was previously reported in Asteraceae (41), Cucurbitaceae (42, 43), and Poaceae (26, 44). Survival on barley roots of seed-borne bacteria occurred in the nongrowing part of the root system but not in emerging roots that are colonized principally by soilborne bacteria (44). While we did not investigate the fate of seed-borne microorganisms in different parts of the root system of *B. napus*, the same proportions of seed-borne taxa were detected within stems and roots. Hence, the weak transmission of seed-borne taxa to seedlings was not a consequence of microhabitat heterogeneity. Overall, these results highlighted that the soil microbiota takes precedence over the seed microbiota.

### Seedling microbiota assembly is mainly driven by soil diversity but not by seed microbiota.

The soil recolonization approach employed in this work resulted in two soils with contrasting levels of bacterial and fungal diversity. This modification of soil microbial diversity also impacted the functional diversity and, ultimately, some soil physicochemical parameters. Changes in NH_4_^+^ and NO_3_^−^ concentrations and pH between high- (S1) and low-diversity (S2) soils were indeed monitored. The switch in the net balance between mineral nitrogen forms, from dominant nitrate forms in the soil S1 to dominant ammonium forms in soil S2, is in agreement with a previous observation in another soil dilution experiment (45). A lower NO_3_^−^ amount was consistent with a reduced abundance of bacterial and archaeal ammonia oxidizers involved in the nitrification process in the S2 soil. The nitrification process is associated with pH reduction prior to the NO_2_^−^ accumulation period, with a potential decrease of 0.75 pH units as observed in the soil S1 (46). Hence, the influence of soil on seedling microbiota structure was not due solely to the initial level of microbial diversity but also to local changes in physicochemical parameters. Nevertheless, the initial soil microbial diversity was found to be the key driver in the assembly of stem and root microbiota.

Contrary to what has been observed in the soil, we did not detect any significant impact of the initial seed microbiota composition on the overall structure of the stem and root microbiota. This observation reflected not only a low transmission of seed-borne taxa to roots and stems of *B. napus* but also a weak, if not absent, historical contingency. Historical contingency is mediated by priority effects that correspond to the impact of species on one another depending on their order of arrival within the local community (29). The importance of historical contingency in the assembly of the plant microbiota was recently highlighted in wheat, where the identity of seed-transmitted fungal taxa modified the colonization of roots by dark septate endophytes, 3 weeks following germination (19). While we cannot conclude there is an absence of priority effects between microbial species within the root and stem microbial communities of *B. napus*, historical contingency was not promoted during seedling community assembly in this study.

The outcome of the seed and soil microbiota encounter was clearly asymmetric because it largely favored the dominance of soil taxa in the assembly of the seedling microbiota. A symmetrical outcome would have been characterized by an equal contribution of the two initial seed and soil communities to the resultant seedling community. The encounter between two microbial communities and the characteristics of their dynamic assembly is currently being reexamined under the community coalescence framework (36, 47, 48). Although seed microbial taxa can be considered plant residents that are adapted to available niches (49), the successful invasion of root and stem by soilborne taxa was undoubtedly facilitated by their population sizes. We conclude that the results were due to a soil microbial mass effect rather than a resident seed advantage. We observed a higher genetic diversity in our soils than in seed; therefore, the dominance of soil-associated microbial communities in seedlings could alternatively be explained by the larger amount of phylogenetic diversity in soil than in seeds. Higher phylogenetic diversity could indeed increase the functional capabilities of soil-associated microbial communities and then limit niche overlap between microbial entities (50). The niche changes occurring for seed at sowing due to moisture were the same in soils S1 and S2, and those due to soil nutrients depended on soils S1 and S2. Soil microbiota had a great influence on the plant microbiota composition, since 70% and 30% of soilborne bacterial and fungal taxa, respectively, were detected in seedlings. This high percentage was deduced from soil taxa on all seedlings, whereas these taxa were probably not all present on each plant. The levels of diversity of the initial seed and soil pools were, however, not directly correlated with the number of seedling-transmitted ASVs (Fig. 5). In other words, the diversity of the regional species pool (i.e., soils and seeds) was not a good predictor of the diversity of local communities (i.e., seedlings).

### Seedling microbiota is primarily composed of initially rare and intermediate-abundance taxa in source communities.

The initial abundance of microbial taxa in the regional species pool was not positively correlated with their seedling transmission. This was particularly marked for bacterial ASVs, where rare seed-borne taxa and intermediate soilborne taxa were mostly transmitted to the seedling (Fig. 6). A conceptual figure summarizes the outcome of the different encounter experiments (see Fig. S4 in the supplemental material). The selection of seed-borne rare taxa by the plant was not expected. Indeed, seed-to-seedling transmission of phytopathogenic bacterial strains is dependent on a minimum bacterial population size (21, 51). An increase in the abundance of rare taxa in seedlings could be explained by several attributes, including more favorable environmental conditions for their growth and awakening from dormancy (52). Many seed-borne bacteria enter a viable but nonculturable (VBNC) state (53–55). A nutrient-rich environment, such as the spermosphere (56), can elicit the resuscitation of VBNC cells (57). In any case, the rare taxa that are selected on seedlings must carry specific genetic determinants that are responsible for their higher fitness that deserve to be investigated further. The emergence of rare taxa seems to be a shared coalescence outcome between different ecosystems, i.e., for the mixing of freshwater and marine microbiome (48), the unequal mixing of two soils with different physicochemical and microbial compositions (58), or the mixing of soils for the outcome of rhizobial communities on rooibos nodules (59). Rare taxa becoming abundant can provide essential or new functions in nutrient cycling or plant growth, which replace or compensate for the function deficiency of abundant species (52, 60).

10.1128/mSystems.00446-21.4 FIG S4 Asymmetric outcome of coalescence of seed and soil microbiota during early seedling growth: relative abundance and transmission of bacterial (A) and fungal (B) taxa. Download FIG S4, DOCX file, 0.2 MB. Copyright © 2021 Rochefort et al. 2021 Rochefort et al. https://creativecommons.org/licenses/by/4.0/ This content is distributed under the terms of the Creative Commons Attribution 4.0 International license.

Interestingly, abundant taxa in the source pool (seed or soil) almost never became abundant in roots and stems. If these taxa (e.g., Pantoea agglomerans) are well adapted to the mature seed habitat, notably, the low-osmotic condition, they appear to be less competitive than rare seed-borne taxa for seedling colonization. Conversely, the best transmission of abundant and intermediate soilborne taxa to seedlings likely combines a soil microbiota mass effect and a plant selective process among soil bacteria that are more adapted to root and stem habitats.

### Conclusion.

In conclusion, we highlighted that the outcome of seed and soil microbiota encounters is a clear dominance of soil microbiota in seedlings. The soil was an important driver of the structure of the seedling microbiota. Among the different soil properties, microbial diversity had the highest influence on seedling microbiota composition in comparison to that of pH and NH_4_^+^ and NO_3_^−^ concentrations. Our approach enabled us to quantify the relative contribution of seed-borne and soilborne taxa to seedling microbiota assembly and provide an estimation of transmission success of each source microbiota. Of all soil taxa detected, only 8% to 32% were able to colonize seedlings, while the proportion was 0.8% to 1.4% for seed-borne taxa, which indicates a strong selection during seedling microbiota assembly. We demonstrate a high transmission of rare seed-borne and intermediate-abundance soilborne taxa to seedlings, which is also a key feature of this encounter (Fig. S4). These results provide an important foundation for the development of plant microbiome engineering through the modification of native seed or soil microbiota. Particularly, we encourage future work using natural or synthetic communities to integrate rare microbial taxa, as we showed that focusing only on abundant taxa in the seed and soil communities is not informative to understand future seedling microbiota composition. Further studies are needed to understand the ecological processes involved in the encounter of seed and soil and how direct inoculation of microorganisms or modification of environmental conditions might alter its outcome.

## MATERIALS AND METHODS

### Soil preparation.

In 2014, soil was sampled at a depth of 10 to 30 cm in a 20-year wheat experimental plot (La Gruche, Pacé, France, 48°08′24.5′′N, 1°48′01.0′′W). Soil was sterilized and recolonized with two levels of soil microbial diversity according to the experimental procedure described in reference 61. In short, a fraction of the soil sample was ground and sieved at 2 mm. Three subsamples (900 g each) were soaked in 8 liters of sterile water. The resulting initial soil suspensions were 1:10 serially diluted in sterile water up to 10^−6^. Another fraction (∼80 kg) of the soil sample was ground, sieved at 4 mm, and mixed with one-third washed sand. This soil was dispatched in 2.5-kg bags and gamma irradiated (35 kGy; Ionisos, France). Each bag containing the same sterilized soil was inoculated with 320 ml of initial or 10^−6^ diluted soil suspensions, therefore resulting in two soils with high (S1) and low (S2) microbial diversity, respectively. The soil recolonization was repeated 3 times (A, B, and C). The bags were mixed and aerated under sterile conditions twice a week during the 39 incubation days at 20°C to homogenize gaseous exchanges and recolonization. Microbial recolonization dynamics of soil was monitored by sampling 30 g of soil at several times. For bacteria, each sample was serial diluted and plated on one-tenth strength tryptic soy agar (17 g·liter^−1^ tryptone, 3 g·liter^−1^ soybean peptone, 2.5 g·liter^−1^ glucose, 5 g·liter^−1^ NaCl, 5 g·liter^−1^ K_2_HPO_4_, and 15 g·liter^−1^ agar) supplemented with nystatin (0.025 g·liter^−1^), and the number of CFU was measured after 3 days of incubation at 27°C. For fungi, according to the pour plate method, serially diluted samples were mixed with molten acid malt agar (10 g·liter^−1^ malt extract, 15 g·liter^−1^ agar, and 0.25 g·liter^−1^ citric acid) supplemented with streptomycin (0.15 g·liter^−1^) and penicillin (0.075 g·liter^−1^), and the number of CFU was measured after 7 days of incubation at 20°C. After 39 days of incubation, a portion of each soil (S1 and S2) in the three independent repetitions (A, B, and C) was collected and stored at −80°C until DNA extraction. Organic and mineral compositions and pH of these samples were analyzed (INRAE, Arras, France). Differences between soil composition for pH and nitric and ammoniacal nitrogen were considered significant with *t* tests at a *P* value of <0.1.

### Sowing and sampling.

After 39 days of soil incubation, individual pots were filled with a 5-mm layer of sterile vermiculite and 80 g of soil of high or low microbial diversity. Soils were saturated with tap water by subimbibition 1 day before sowing in order to reach approximately 80% humidity (retention capacity) on the day of sowing. During the experiment, plants were watered twice (at 5 and 12 days) with tap water.

Seed samples from two genotypes of *Brassica napus* (Boston and Major) collected during two consecutive years (Y1 and Y2) were selected for this work, resulting in four contrasting initial seed microbiota used for the encounter experiment. At d0, seeds of each genotype and year were individually sown at a depth of 5 mm and grown under controlled conditions (14-h day/10-h night period, 20°C). Seven days (d07) and 14 days (d14) after sowing, 10 and 20 plants were sampled per modality (2 genotypes × 2 harvesting years × 2 soils), respectively. The objective for taking two time points (two plant developmental stages) was to determine if the first event of assembly was transient or kept during the early growth. Roots were cut from stems and gently soaked 10 s in sterile water to remove residual soil. Otherwise, stems were size equalized 2 cm from the root basis. Therefore, the resulting sampled root habitat was composed of the inner root and rhizoplane, and the stem habitat was composed of inner stem and stem surface. The 10 (d07) or 20 (d14) roots and stems were pooled separately and stored as root and stem compartments at −80°C until DNA extraction. The experiment was conducted in three independent repeats (A, B, and C) for each soil (S1 and S2).

### Microbial DNA sample preparation.

Seed samples were prepared for extraction as previously detailed (32). Briefly, seeds were soaked in phosphate-buffered saline (PBS; Sigma-Aldrich) supplemented with Tween 20 (0.05% [vol/vol]; Sigma-Aldrich) for 2 h 30 min at 4°C. Soil (250 mg per repeat A, B, and C), root, and stem samples were lyophilized before DNA extraction. Samples were mixed with 1-mm and 3-mm beads and crushed 2 × 30 s at 5 m/s with FastPrep (MP Biomedicals). DNA extraction was performed with the DNeasy PowerSoil HTP 96 kit (Qiagen) according to the manufacturer’s procedure.

### Library construction and sequencing.

Amplicon libraries were constructed with the primer sets gyrB_aF64/gyrB_aR553 (250 bp with length polymorphism [241 to 268]) (25) and ITS1F/ITS2 (62), according to the experimental procedure previously described (32). A blank extraction kit control and a PCR negative control were included in each PCR plate. Amplicon libraries were mixed with 10% PhiX and sequenced with two MiSeq reagent kit v3 600 cycles (paired-end sequencing).

### Sequence processing.

Primer sequences were removed with cutadapt version 1.8 (63). Fastq files were processed with DADA2 version 1.6.0 (64) using the following parameters: truncLen, c(190, 200); maxN, 0; maxEE, c(1); truncQ, 5 for *gyrB* reads. ITS1 reads were processed with the same parameters, except that reads were not trimmed to a fixed length and trunQ was set to 2. Chimeric sequences were identified and removed with the removeBimeraDenovo function of DADA2. Taxonomic affiliations of amplicon sequence variants (ASV) were performed with a naive Bayesian classifier (65) implemented in DADA2. ASVs derived from *gyrB* reads were classified with an in-house *gyrB* database (train_set_gyrB_v4.fa.gz), available upon request. ASVs derived from ITS1 reads were classified with the UNITE v7.1 fungal database (66).

### Microbial community analyses.

Analyses of diversity were conducted with the R package phyloseq version 1.26.0 (67). Since the primer set gyrB_aF64/gyrB_aR553 can sometimes coamplify *parE*, a paralog of *gyrB*, the *gyrB* taxonomic training data also contained *parE* sequences. Hence, ASVs affiliated with *parE* or unclassified at the phylum level were removed. Sequences were aligned with DECIPHER 2.14.0 (68), and neighbor-joining phylogenetic trees were constructed with phangorn 2.5.5 (69). Identification of sequence contaminants was assessed with decontam version 1.4 (70) using the “prevalence” method at a threshold of 0.1. Fungal ASVs unassigned at the phylum level were removed. For the ITS1 data set, we created a hybrid-gene phylogenetic tree (i.e., ghost tree) (71) based on the 18S rRNA gene sequence database (Silva 132) and ITS database (UNITE v8) in order to perform phylogenetic analyses.

Data were normalized based on sequencing depth. For soil analyses, data were rarefied at 30,000 and 25,000 reads per sample for *gyrB* and ITS1, respectively. For plant (root and stem) analyses, data were rarefied at 1,000 reads per sample for *gyrB* and ITS1 (see Fig. S5 in the supplemental material), a validated choice to keep an optimal number of reliable samples. We did not apply a rarefaction filter for the analysis of presence/absence of taxa to investigate taxa detection and their transmission. Faith’s phylogenetic diversity was calculated on *gyrB* and ITS1 data sets with the R package picante version 1.8 (72). Differences in alpha-diversity estimators were assessed with analysis of variance. Differences were considered significant at a *P* value of <0.05.

10.1128/mSystems.00446-21.5 FIG S5 Rarefaction curves of bacterial and fungal richness in soil (A and B), seed (C and D), and root and stem (E and F) samples. Species richness represents the number of bacterial or fungal ASVs, and sample size represents the number of reads. The vertical line represents the chosen rarefaction threshold. Download FIG S5, DOCX file, 0.2 MB. Copyright © 2021 Rochefort et al. 2021 Rochefort et al. https://creativecommons.org/licenses/by/4.0/ This content is distributed under the terms of the Creative Commons Attribution 4.0 International license.

Changes in microbial community composition were assessed on log_10_ + 1-transformed values with the Bray-Curtis (BC) index. Principal-coordinate analysis (PCoA) was used for ordination of the BC index. The homogeneity of group dispersions was assessed with the betadisper function of vegan 2.5.3 (73). Differences in dispersion between groups were assessed with a Wilcoxon nonparametric test. To quantify the relative contributions of seed microbiota, soil diversity, development stage, habitat, and initial soil physicochemical parameters in microbial community profiles, permutational multivariate analysis of variance (PERMANOVA) (74) was performed with the function adonis2 of the R package vegan 2.5.3 (73). The relative abundances of ASVs belonging to ammonia-oxidizing bacteria (AOB) and nitric-oxidizing bacteria (NOB) according to their taxonomic classification were investigated in soils S1 (high diversity) and S2 (low diversity). These include *Nitrosospira* sp. and *Nitrosovibrio* sp. for AOB and *Nitrobacter* sp. and *Nitrospira* sp. for NOB (75, 76). Differences in relative abundances of AOB and NOB were considered significant with *t* test at a *P* value of <0.08 and a *P* value of <0.05, respectively.

### Transmission analyses.

A prevalence matrix of common ASVs between seeds, soils, roots, and stems was constructed on nonrarefied data to have the most exhaustive presence-absence analysis of all taxa. Owing to the microbial variability between individual seeds, an ASV was recorded as present if detected in at least one of the 3 seed sample repetitions. Visualization of bacterial and fungal ASVs shared between biological habitats or specific to one was assessed with the R package UpSetR 1.4.0 (77).

### Data availability.

The data sets supporting the conclusions of this article are available in the European Nucleotide Archive under the accession number PRJEB41004. R code and files for all microbiota analyses, statistics, and figure generation are available on GitHub (https://github.com/arochefort/Seed_Soil_Coalescence).
