# Supplementary material for: Transmission of Seed and Soil Microbiota to Seedling
Source: mSystems. 2021 Jun 8;6(3):e00446-21. doi: 10.1128/mSystems.00446-21 (PMC8269233; doi:10.1128/mSystems.00446-21)
Supplement: TABLE S1 [file msystems.00446-21-st001.docx]

| Bacteria |  |  |  |  |
| --- | --- | --- | --- | --- |
|  | **ASV** | **Order** | **Species** | **Relative abundance in seed** |
| root & stem | ASV314 | Micrococcales | *Arthrobacter sp.* | Rare |
|  | ASV448 | Pseudomonadales | *Pseudomonas sp.* | Rare |
|  | ASV458 | Micrococcales | *Terrabacter sp.* | Rare |
|  | ASV480 | Micrococcales | *Arthrobacter sp.* | Rare |
|  | ASV546 | Sphingomonadales | Erythrobacteraceae | Rare |
|  | ASV623 | Rhodobacterales | *Paracoccus yeei* | Rare |
|  | ASV757 | Sphingomonadales | *Blastomonas sp.* | Intermediate |
|  | ASV251 | Rhizobiales | *Afipia sp.* | Abundant |
| root only | ASV1000 | Micrococcales | *Arthrobacter sp.* | Rare |
|  | ASV1119 | Propionibacteriales | *Nocardioides sp.* | Rare |
|  | ASV4636 | Rhodobacterales | *Paracoccus sp.* | Rare |
| stem only | ASV273 | Bacillales | *Bacillus megaterium* | Rare |
|  | ASV334 | Xanthomonadales | *Stenotrophomonas rhizophila* | Rare |
|  | ASV419 | Burkholderiales | *Achromobacter sp.* | Rare |
|  | ASV651 | Micrococcales | *Arthrobacter sp.* | Rare |
|  | ASV2583 | Micrococcales | *Microbacterium sp.* | Rare |
|  | ASV479 | Propionibacteriales | *Cutibacterium acnes* | Intermediate |
|  | ASV1502 | Pseudomonadales | *Pseudomonas lurida* | Intermediate |
|  | ASV2520 | Rhizobiales | *Hyphomicrobium sp.* | Intermediate |

| Fungi |  |  |  |  |
| --- | --- | --- | --- | --- |
|  | **ASV** | **Order** | **Species** | **Relative abundance in seed** |
| root & stem | ASV170 | Hypocreales | *Gibberella avenacea* | Rare |
|  | ASV42 | Pleosporales | *Alternaria sp.* | Intermediate |
|  | ASV3 | Pleosporales | *Alternaria infectoria* | Abundant |
|  | ASV7 | Capnodiales | *Cladosporium delicatulum* | Abundant |
| stem only | ASV11 | Pleosporales | *Alternaria infectoria* | Abundant |
|  | ASV20 | Pleosporales | *Alternaria infectoria* | Abundant |
|  | ASV22 | Pleosporales | *Alternaria infectoria* | Abundant |
