# Supplementary material for: Transmission of Seed and Soil Microbiota to Seedling
Source: mSystems. 2021 Jun 8;6(3):e00446-21. doi: 10.1128/mSystems.00446-21 (PMC8269233; doi:10.1128/mSystems.00446-21)
Supplement: TABLE S2 [file msystems.00446-21-st002.docx]

| **Bacteria** |  |  |  |  |  |
| --- | --- | --- | --- | --- | --- |
|  | **ASV** | **Order** | **Species** | **Relative abundance in soil** |  |
| **root & stem** | ASV5 | Bacillales | *Fictibacillus arsenicus* | Intermediate |  |
|  | ASV8 | Burkholderiales | *Massilia sp.* | Intermediate |  |
|  | ASV13 | Pseudomonadales | *Pseudomonas sp.* | Intermediate |  |
|  | ASV20 | Rhizobiales | *Bosea sp.* | Intermediate |  |
|  | ASV22 | Rhizobiales | *Devosia sp.* | Intermediate |  |
|  | ASV30 | Propionibacteriales | *Nocardioides sp.* | Intermediate |  |
|  | ASV38 | Caulobacterales | *Caulobacter sp.* | Intermediate |  |
|  | ASV42 | Sphingomonadales | *Sphingopyxis sp.* | Intermediate |  |
|  | ASV49 | Sphingomonadales | *Sphingomonas sp.* | Intermediate |  |
|  | ASV51 | Rhizobiales | *Afipia sp.* | Intermediate |  |
|  | ASV54 | Burkholderiales | unclassified | Intermediate |  |
|  | ASV66 | Nitrosomonadales | *Methylobacillus sp.* | Intermediate |  |
|  | ASV4 | Burkholderiales | *Massilia sp.* | Abundant |  |
|  | ASV24 | Propionibacteriales | *Nocardioides sp.* | Abundant |  |
|  | ASV35 | Sphingomonadales | *Sphingomonas pruni* | Abundant |  |
| **root only** | ASV33 | Burkholderiales | Oxalobacteraceae | Intermediate |  |
|  | ASV36 | Rhizobiales | *Afipia sp.* | Intermediate |  |
|  | ASV43 | Burkholderiales | *Massilia sp.* | Intermediate |  |
|  | ASV68 | Sphingomonadales | Sphingomonadaceae | Intermediate |  |
|  | ASV71 | Burkholderiales | Comamonadaceae | Intermediate |  |
|  | ASV106 | Rhizobiales | *Bosea sp.* | Intermediate |  |
|  | ASV138 | Rhizobiales | *Pseudolabrys sp.* | Intermediate |  |
|  | ASV141 | Sphingomonadales | *Sphingopyxis sp.* | Intermediate |  |
|  | ASV145 | Propionibacteriales | *Aeromicrobium sp.* | Intermediate |  |
|  | ASV155 | Bacteroidetes | unclassified | Intermediate |  |
|  | ASV181 | Micrococcales | *Microbacterium sp.* | Intermediate |  |
|  | ASV227 | Burkholderiales | *Variovorax sp.* | Intermediate |  |
|  | ASV274 | Caulobacterales | *Phenylobacterium sp.* | Intermediate |  |
|  | ASV290 | Caulobacterales | *Caulobacter sp.* | Intermediate |  |
|  | ASV23 | Burkholderiales | *Massilia sp.* | Abundant |  |
|  | ASV39 | Sphingomonadales | *Sphingopyxis sp.* | Abundant |  |
| **stem only** | ASV359 | Alphaproteobacteria | unclassified | Rare |  |
|  | ASV12 | Pseudomonadales | *Pseudomonas fluorescens* | Intermediate |  |
|  | ASV14 | Pseudomonadales | *Pseudomonas sp.* | Intermediate |  |
|  | ASV26 | Burkholderiales | *Achromobacter sp.* | Intermediate |  |
|  | ASV37 | Burkholderiales | unclassified | Intermediate |  |
|  | ASV62 | Xanthomonadales | Xanthomonadaceae | Intermediate |  |
|  | ASV67 | Pseudomonadales | *Pseudomonas sp.* | Intermediate |  |
|  | ASV87 | Xanthomonadales | Xanthomonadaceae | Intermediate |  |
|  | ASV104 | Burkholderiales | unclassified | Intermediate |  |
|  | ASV128 | Pseudomonadales | *Pseudomonas moraviensis* | Intermediate |  |
|  | ASV137 | Rhizobiales | Hyphomicrobiaceae | Intermediate |  |
|  | ASV148 | Rhizobiales | *Devosia sp.* | Intermediate |  |
|  | ASV229 | Chitinophagales | Chitinophagaceae | Intermediate |  |
|  | ASV19 | Pseudomonadales | *Pseudomonas sp.* | Abundant |  |
|  | ASV34 | Pseudomonadales | *Pseudomonas fluorescens* | Abundant |  |
|  | ASV48 | Pseudomonadales | *Pseudomonas fluorescens* | Abundant |  |

| **Fungi** |  |  |  |  |
| --- | --- | --- | --- | --- |
|  | **ASV** | **Order** | **Species** | **Relative abundance in soil** |
| **root & stem** | ASV48 | Hypocreales | Nectriaceae | Rare |
|  | ASV55 | Hypocreales | *Dactylonectria novozelandica* | Rare |
|  | ASV63 | Hypocreales | *Neocosmospora rubicola* | Rare |
|  | ASV343 | Hypocreales | *Bionectria rossmaniae* | Rare |
|  | ASV477 | Diversisporales | *Entrophospora sp.* | Rare |
|  | ASV4 | Pezizales | *Peziza ostracoderma* | Intermediate |
|  | ASV7 | Capnodiales | *Cladosporium delicatulum* | Intermediate |
|  | ASV13 | Hypocreales | *Fusarium sp.* | Intermediate |
|  | ASV18 | Hypocreales | *Clonostachys rosea* | Intermediate |
|  | ASV29 | Hypocreales | *Fusicolla merismoides* | Intermediate |
|  | ASV34 | Trichosporonales | *Cutaneotrichosporon sp.* | Intermediate |
|  | ASV40 | Hypocreales | *Fusicolla aquaeductuum* | Intermediate |
|  | ASV47 | Hypocreales | *Acremonium curvulum* | Intermediate |
|  | ASV49 | Trichosporonales | *Trichosporon porosum* | Intermediate |
|  | ASV59 | Hypocreales | *Fusarium solani* | Intermediate |
|  | ASV62 | Hypocreales | *Volutella ciliata* | Intermediate |
|  | ASV96 | Hypocreales | *Gibberella intricans* | Intermediate |
|  | ASV141 | Hypocreales | *Metarhizium marquandii* | Intermediate |
|  | ASV170 | Hypocreales | *Gibberella avenacea* | Intermediate |
|  | ASV2 | Hypocreales | Nectriaceae | Abundant |
|  | ASV5 | Hypocreales | *Fusarium sp.* | Abundant |
|  | ASV6 | Mortierellales | *Mortierella elongata* | Abundant |
|  | ASV8 | Mortierellales | *Mortierella elongata* | Abundant |
|  | ASV30 | Sordariales | *Trichocladium asperum* | Abundant |
|  | ASV32 | Hypocreales | *Metarhizium marquandii* | Abundant |
|  | ASV35 | Trichosporonales | *Apiotrichum gracile* | Abundant |
| **root only** | ASV613 | Microascales | uncassified | Rare |
|  | ASV23 | Hypocreales | *Trichoderma atroviride* | Intermediate |
|  | ASV52 | Sordariales | *Chaetomium piluliferum* | Intermediate |
|  | ASV75 | Tremellales | uncassified | Intermediate |
|  | ASV146 | Hypocreales | *Clonostachys sp.* | Intermediate |
|  | ASV153 | Hypocreales | *Trichoderma velutinum* | Intermediate |
|  | ASV301 | Pezizales | *Peziza ostracoderma* | Intermediate |
|  | ASV17 | Mortierellales | *Mortierella alpina* | Abundant |
|  | ASV26 | Mortierellales | *Mortierella alpina* | Abundant |
| **stem only** | ASV11 | Pleosporales | *Alternaria infectoria* | Rare |
|  | ASV268 | Hypocreales | *Bionectria rossmaniae* | Intermediate |
