# Supplementary figures and images for: Transmission of Seed and Soil Microbiota to Seedling
Source: mSystems. 2021 Jun 8;6(3):e00446-21. doi: 10.1128/mSystems.00446-21 (PMC8269233; doi:10.1128/mSystems.00446-21)

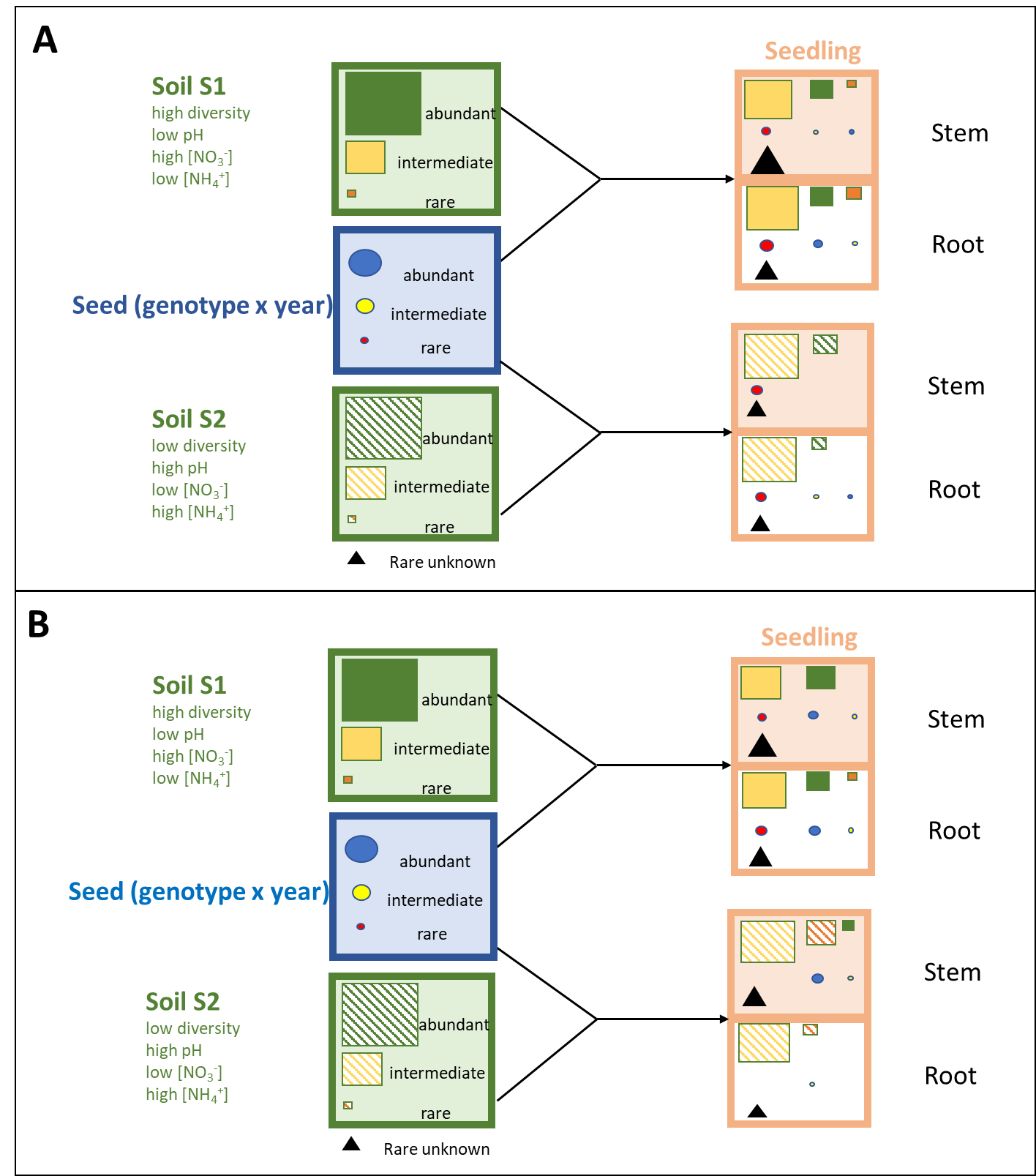

Supplement: FIG S4 [file msystems.00446-21-sf004.docx]
