# Supplementary material for: Transmission of Seed and Soil Microbiota to Seedling
Source: mSystems. 2021 Jun 8;6(3):e00446-21. doi: 10.1128/mSystems.00446-21 (PMC8269233; doi:10.1128/mSystems.00446-21)

A - Bacteria

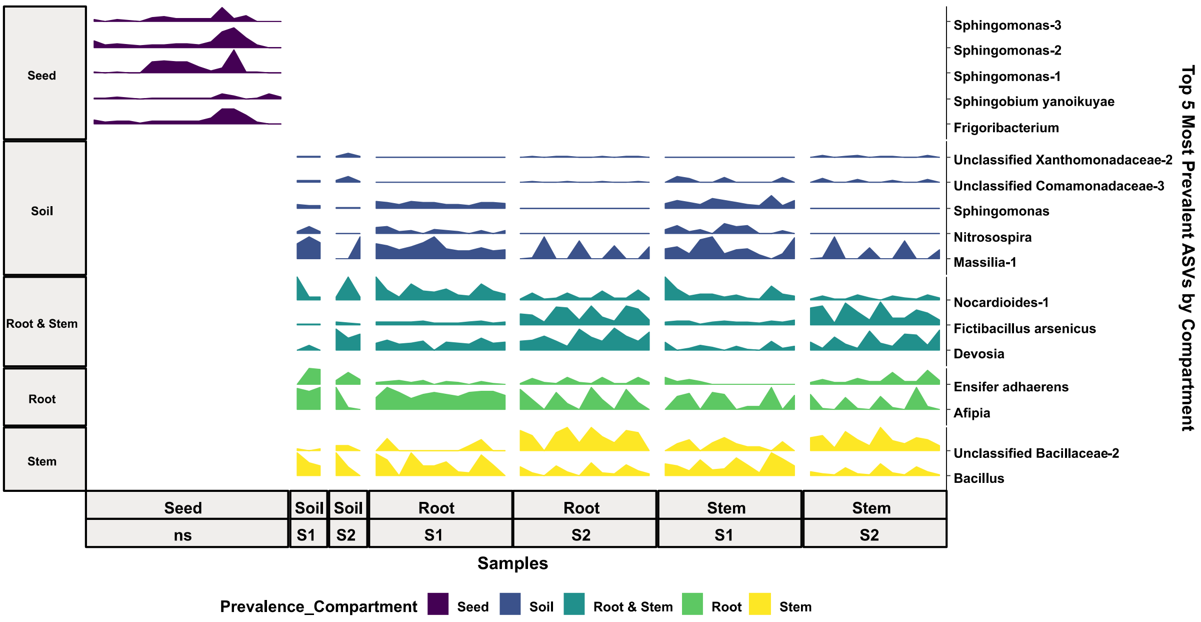

B - Fungi

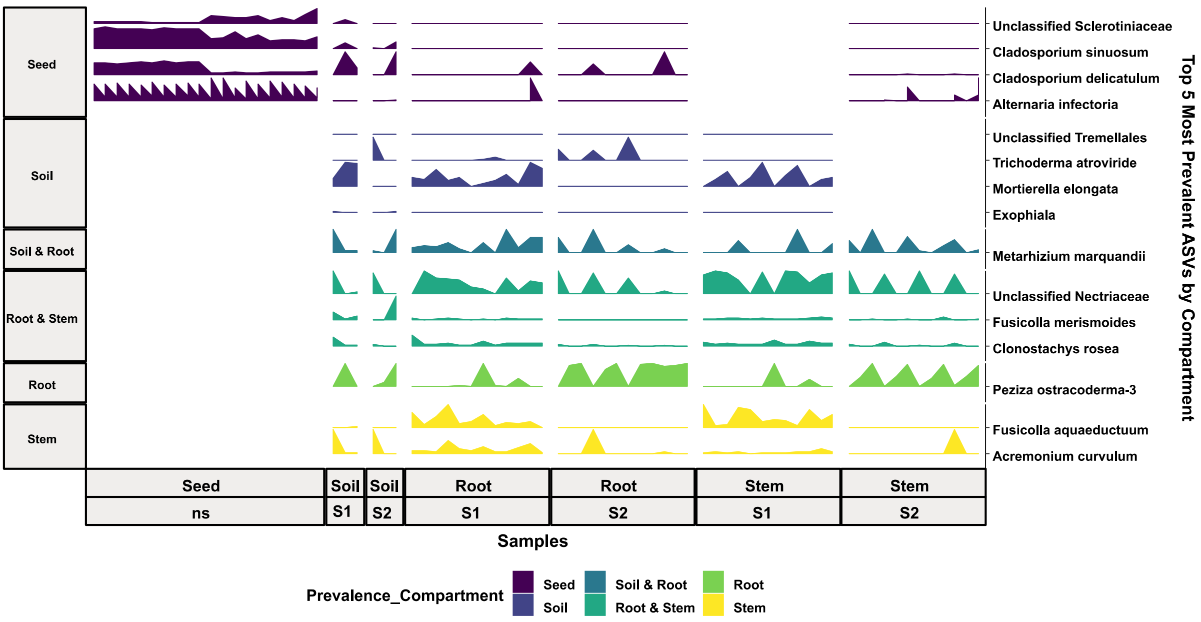

Supplement: Supplementary file 4 [file msystems.00446-21-sf001.docx]
