# Supplementary material for: Transmission of Seed and Soil Microbiota to Seedling
Source: mSystems. 2021 Jun 8;6(3):e00446-21. doi: 10.1128/mSystems.00446-21 (PMC8269233; doi:10.1128/mSystems.00446-21)
Supplement: Supplementary file 5 [file msystems.00446-21-sf002.docx]

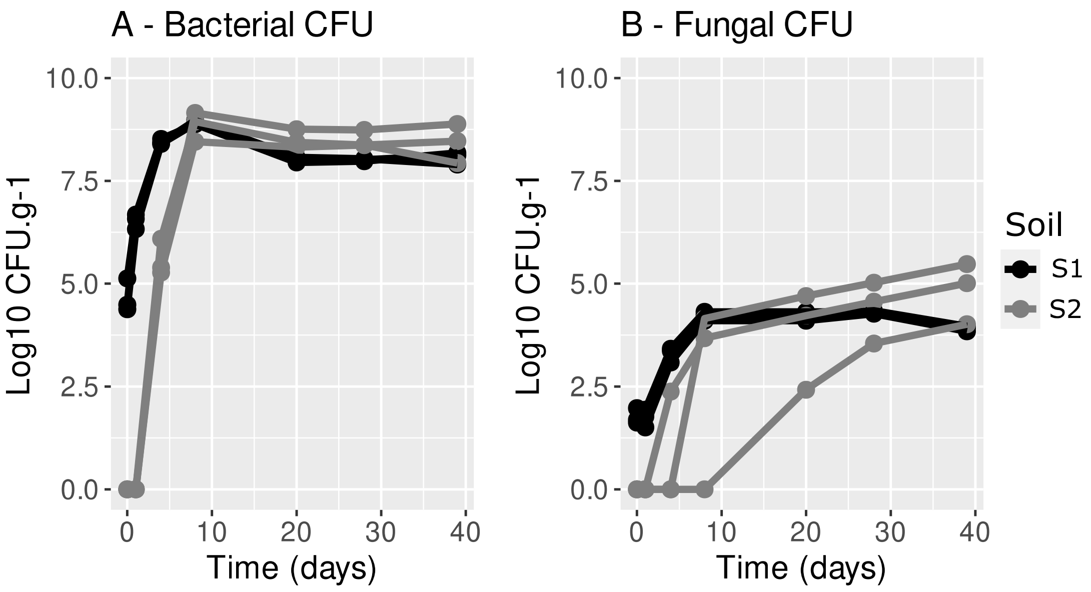
A - Bacterial CFU
B - Fungal CFU
Log10 CFU.g-1
Time (days)
10.0
7.5
5.0
2.5
0.0
0
10
20
30
40
Soil
S1
S2
