# Supplementary material for: Transmission of Seed and Soil Microbiota to Seedling
Source: mSystems. 2021 Jun 8;6(3):e00446-21. doi: 10.1128/mSystems.00446-21 (PMC8269233; doi:10.1128/mSystems.00446-21)
Supplement: Supplementary file 6 [file msystems.00446-21-sf003.docx]

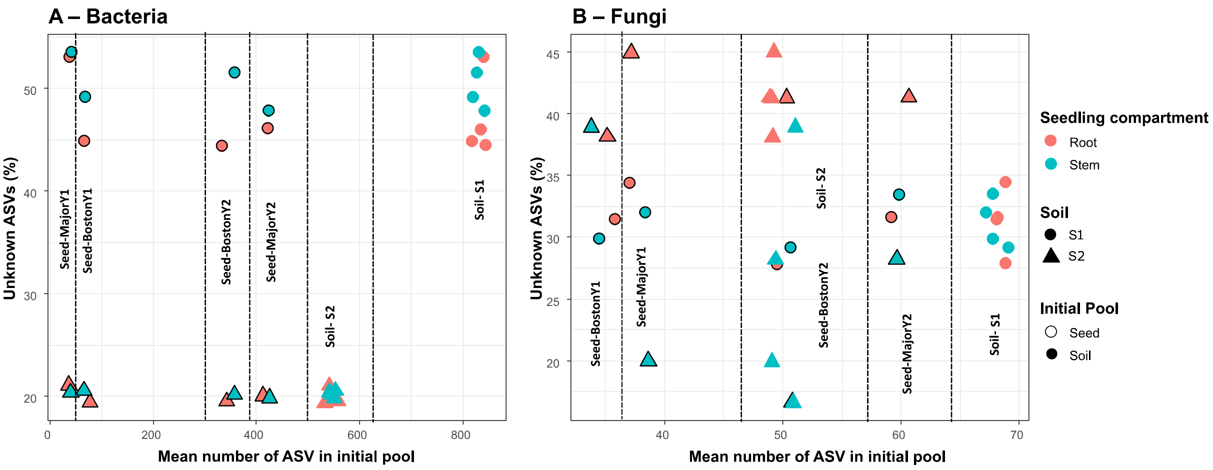

A – Bacteria
B – Fungi
Unknown ASVs (%)
Mean number of ASV in initial pool
Seed-MajorY1
Seed-BostonY1
Seed-BostonY2
Seed-MajorY2
Soil- S2
Soil- S1
Seedling compartment
Root
Stem
Soil
S1
S2
Initial Pool
Seed
Soil
