# Supplementary material for: Transmission of Seed and Soil Microbiota to Seedling
Source: mSystems. 2021 Jun 8;6(3):e00446-21. doi: 10.1128/mSystems.00446-21 (PMC8269233; doi:10.1128/mSystems.00446-21)
Supplement: Supplementary file 7 [file msystems.00446-21-sf005.docx]

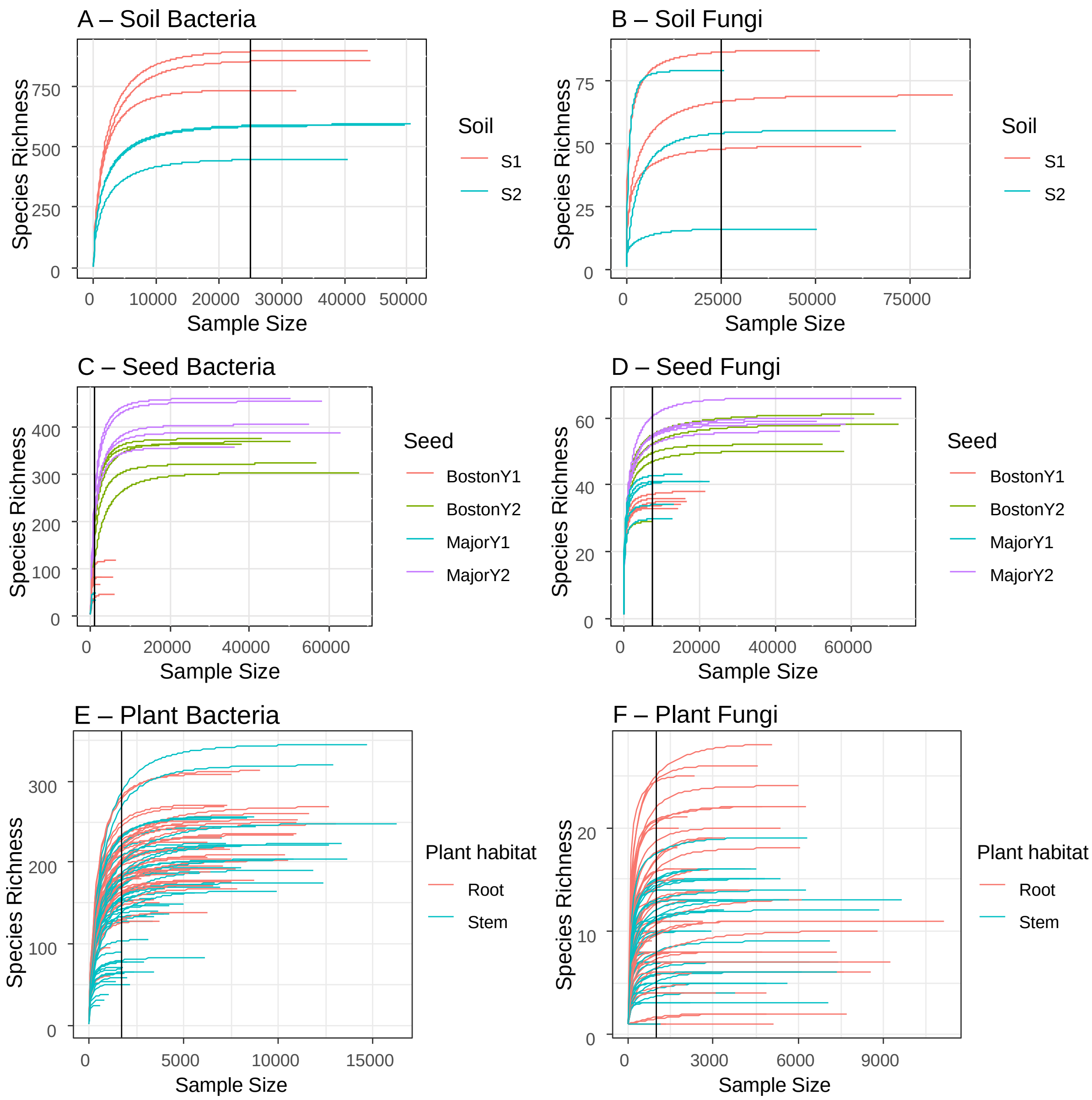

A – Soil Bacteria
B – Soil Fungi
C – Seed Bacteria
D – Seed Fungi
E – Plant Bacteria
F – Plant Fungi
Species Richness
Sample Size
Soil
S1
S2
Seed
BostonY1
BostonY2
MajorY1
MajorY2
Plant habitat
Root
Stem
